# Unraveling the kinetic diversity of microbial 3-dehydroquinate dehydratases of shikimate pathway

**DOI:** 10.1186/s13568-014-0087-y

**Published:** 2015-02-01

**Authors:** Chang Liu, Yi-Ming Liu, Qing-Lan Sun, Cheng-Ying Jiang, Shuang-Jiang Liu

**Affiliations:** State Key Laboratory of Microbial Resources, Chinese Academy of Sciences, Beijing, 100101 China; Environmental Microbiology Research Center, Chinese Academy of Sciences, Beijing, 100101 China; Bioinformatic Center at Institute of Microbiology, Chinese Academy of Sciences, Beijing, 100101 China

**Keywords:** 3-Dehydroquinate dehydratase (DHQase), Kinetic constants, Shikimate pathway, Biobrick, Synthetic biology

## Abstract

3-Dehydroquinate dehydratase (DHQase) catalyzes the conversion of 3-dehydroquinic acid to 3-dehydroshikimic acid of the shikimate pathway. In this study, 3180 prokaryotic genomes were examined and 459 DHQase sequences were retrieved. Based on sequence analysis and their original hosts, 38 DHQase genes were selected for chemical synthesis. The selected DHQases were translated into new DNA sequences according to the genetic codon usage bias by both *Escherichia coli* and *Corynebacterium glutamicum*. The new DNA sequences were customized for synthetic biological applications by adding Biobrick adapters at both ends and by removal of any related restriction endonuclease sites. The customized DHQase genes were successfully expressed in *E. coli*, and functional DHQases were obtained. Kinetic parameters of K_m_, k_cat_, and V_max_ of DHQases were determined with a newly established high-throughput method for DHQase activity assay. Results showed that DHQases possessed broad strength of substrate affinities and catalytic capacities. In addition to the DHQase kinetic diversities, this study generated a DHQase library with known catalytic constants that could be applied to design artificial modules of shikimate pathway for metabolic engineering and synthetic biology.

## Introduction

Shikimate pathway widely exists in microbes and plants, but not animals. This pathway is involved in the synthesis of aromatic amino acids, vitamins, as well as lignin (Herrmann and Weaver 1999; Vanholme et al. 2012). The pathway consists of seven catalytic steps, condensing erythrose-4-phosphate (E4P) and phosphoenolpyruvate (PEP) finally into chorismate. 3-Dehydroquinate dehydratase (DHQase, EC 4.2.1.10) catalyzes the third step, i.e., reversible transformation of DHQ to form 3-dehydroshikimic acid. DHQases belong to the family of lyases, and cleave carbon-oxygen bonds. So far as known, DHQases involve in not only shikimate pathway but also other metabolic processes such as the quinate pathway for synthesis of 4-hydroxybenzoic acid (Giles et al. 1985; Giles et al. 1991). According to their origins and catalytic features, DHQases are classified into either type I or type II. Type I DHQases are heat-liable dimeric (Roszak et al. 2002), and mainly occur in plants and fungi. Type II DHQases are heat-stable dodecameric (Roszak et al. 2002) and widely occur in bacteria for shikimate pathway or in fungi for quinate catabolism (Giles et al. 1991).

Many investigations of DHQases have been focused on their structures and catalytic mechanisms (Blomberg et al. 2009; Bottomley et al. 1996; Devi et al. 2013; Lee et al. 2002; Pan et al. 2012; Roszak et al. 2002), or on structure-based design of inhibitors to DHQase activity (Blanco et al. 2012; 2014; Dias et al. 2011; Peon et al. 2010). These investigations have generated increasing numbers of DHQase structures with high resolution and have significantly advanced the understanding of DHQase catalytic mechanisms (Chaudhuri et al. 1986; Deka et al. 1994; Euverink et al. 1992; Hawkins et al. 1993; Lee et al. 2003; Moore et al. 1993; Roszak et al. 2002; Singh and Christendat 2006). Type I DHQases catalyze the dehydrogenation of DHQ through a *cis*-elimination (Chaudhuri et al. 1991; Gourley et al. 1999; Leech et al. 1995), while type II DHQases take a *trans*-elimination mechanism (Blomberg et al. 2009; Bottomley et al. 1996).

Recent study has revealed that overexpression of DHQase enhanced transformation of quinic acid into shikimic acid in *Gluconobacter oxydans* (Nishikura-Imamura et al. 2014). The kinetic properties of DHQases such as K_m_, V_max_, and k_cat_ are important particularly to design new biocatalysts and to predict the validity and efficiency of newly constructed metabolic networks. So far, only a small number of DHQases from prokaryotes such as *Escherichia coli, Mycobacterium tuberculosis,* and *Streptomyces coelicolor* were characterized for their catalytic properties (Harris et al. 1996; Kleanthous et al. 1992; Moore et al. 1993; Richards et al. 2006; White et al. 1990), and the catalytic and kinetic properties of the majority of microbial DHQases remain still unknown.

In this study, we aimed to investigate the kinetic diversities of DHQases and to expand the toolbox of catalytic parts for synthetic biology. By data-mining of 3180 prokaryotic genomes from NCBI genome database, 459 putative DHQases were targeted. Thirty-eight DHQases were further selected and standardized according to “Biobricks” requirements (Knight 2003; Shetty et al. 2011; Sleight et al. 2010). DHQase kinetic constants were determined with a newly established high-throughput method. Our results showed that DHQases are highly diverse in catalytic kinetics.

## Materials and methods

### Genome data-mining for DHQase genes

The amino acid sequences of the type II DHQase (NP_599670) from *Corynebacterium glutamicum* ATCC13032 and of the type I DHQase (NP_416208) from *E. coli* K-12 were used as seed sequences to retrieve putative DHQase sequences from NCBI genome database with a filter condition of threshold E value ≤ 10^−10^. The retrieved sequences were then screened and redundant copies were removed. To increase the credibility of functional DHQase prediction, the retrieved sequences were further filtered by removal of putative DHQase sequences from which host organisms have incomplete shikimate pathway in their genomes.

### Design and chemical synthesis of DHQase genes

The selected DHQase amino acid sequences were reverse-translated into DNA sequences, and were recoded with referring codon usage bias of *E. coli* and *C. glutamicum*. The obtained DNA sequences were optimized for expression in *E. coli* and *C. glutamicum* by check for RNA secondary structure with software UNAFold (Markham and Zuker 2008). Any predicted secondary structures were eliminated by codon replacements. The new DHQase genes were further customized, by linking to Biobrick adapters (Knight 2003) at both ends. The customized DNA sequences of the DHQases are accessible at http://www.genoportal.org/bbdb under the accession numbers of SBB_00477 ~ SBB_004481, and were chemically synthesized (Sangon Biotech, China), and were cloned in *E. coli*. The DNA sequences of the chemically synthesized DHQase genes were confirmed by DNA sequencing.

### Bacterial strain, plasmids, and growth condition

For cloning and expression of DHQase genes, *E. coli* BL21 (DE3) (TransGen Biotech, China) and plasmid pET28a^+^ (Novagen, Germany) were used. Expression of DHQase genes was induced with 0.1 mmol/L of IPTG when culture reached OD_600nm_ of 0.6-0.8. After addition of IPTG, culture was further incubated at 16°C, overnight. Routine cultivation of *E. coli* proceeded in Luria-Bertani (LB) medium at 37°C and at 200 rpm rotatory shaking. To maintain the stability of pET28a^+^ and its derivatives, kanamycin at final concentration of 100 μg/ml were added into LB medium.

### Preparation of cellular lysates and purification of DHQase proteins

*E. coli* cells were harvested by centrifugation at 10,000 rpm, and suspended in 50 mmol/L Tris–HCl buffer, pH 8.0. After addition of 0.01% (w/v) protease inhibitor cocktail (Amresco, the United States), cell suspension was treated with ultrasonication (work for 3 sec, stop for 5 sec, 100 repeats). The cellular debris was removed by centrifugation at 10,000 rpm for 10 min, and the supernatant was filtered with 0.22 μm filters (Millipore, the United States). The filtered supernatant was collected and DHQase proteins were purified using HisPur™ Ni-NTA Spin Columns Kit (Thermo Scientific, the United States). The purified DHQase proteins were stored at −80°C in 50 mmol/L Tris–HCl (pH 8.0) buffer containing 25% glycerol. All procedures were operated at 4°C unless indicated.

Protein concentrations were determined with Bio-Rad Protein Assay (BIO-RAD, the UK).

Tricine sodium dodecyl sulfate polyacrylamide gel electrophoresis was used to evaluate the DHQase expression and the purity during protein purification. Preparation of gels (4% sample gel and 10% separating gel) and operation of electrophoresis were conducted according to Schägger (2006). Gels were visualized with Coomassie brilliant blue G-250 staining, and were scanned with PC scanner (T68, Founder, China) for imagine analysis.

### DHQase activity assays

A high-throughput method of DHQase activity assay was established in this study. The method has the same principal for measurement as White et al. (1990), but with new systems. Synergy H4 Hybrid Multi-Mode Microplate Reader (BioTek, the UK) and 96 Well UV-Plate (Corning Costar, the United States) were used to monitor the changes of OD_234nm_ of multi-samples. DHQase catalysis was optimized in volume of 100 μL and with various DHQ concentrations (0.08 to 1.0 mmol/L). Kinetic constants K_m_ and V_max_ were calculated according to Lineweaver-Burk plot.

### Sequence alignments and construction of phylogenetic tree

The amino acid sequences of DHQases were retrieved from NCBI genome database. Amino acid sequence alignment of DHQases were performed with MUSCLE (Edgar 2004), and the graphic display of alignments were made by ESPript3.0 (Gouet et al. 1999). The phylogenetic tree of amino acid sequences from all putative DHQases were firstly constructed by Phylip Package (Abdennadher and Boesch 2007) under Linux system with method of maximum likelihood and bootstrap replications of 1000, and further annotated using iTOL v2.2.2 (Letunic and Bork 2011).

## Results

### DHQases are phylogenetically diverse

3180 genomes were explored for putative DHQases, and 459 putative DHQase were targeted, including 128 type I and 331 type II DHQases. Noticeably, all archaeal DHQases were type I. Bacterial genomes carried both type I and type II DHQases, which 79.6% of the bacterial DHQases were type II and 20.4% were type I. Of the 391 genomes explored, 306 genomes (78.3%) harbored only type II DHQases, 60 genomes (15.3%) harbored only type I, and 25 genomes (6.4%) harbored both type I and type II (Table 1).

**Table 1:** **Distribution of type I and type II DHQases in prokaryotic genomes** ^**a**^

**Sources**	**DHQase in a genome**	**Number of genomes**
**Bacteria**	Type I only	60
	Type II only	306
	Both Type I and II	25
**Archaea**	Type I	43

^a^Notes: Redundant genomes representing the same species were removed.

**Figure 1:**
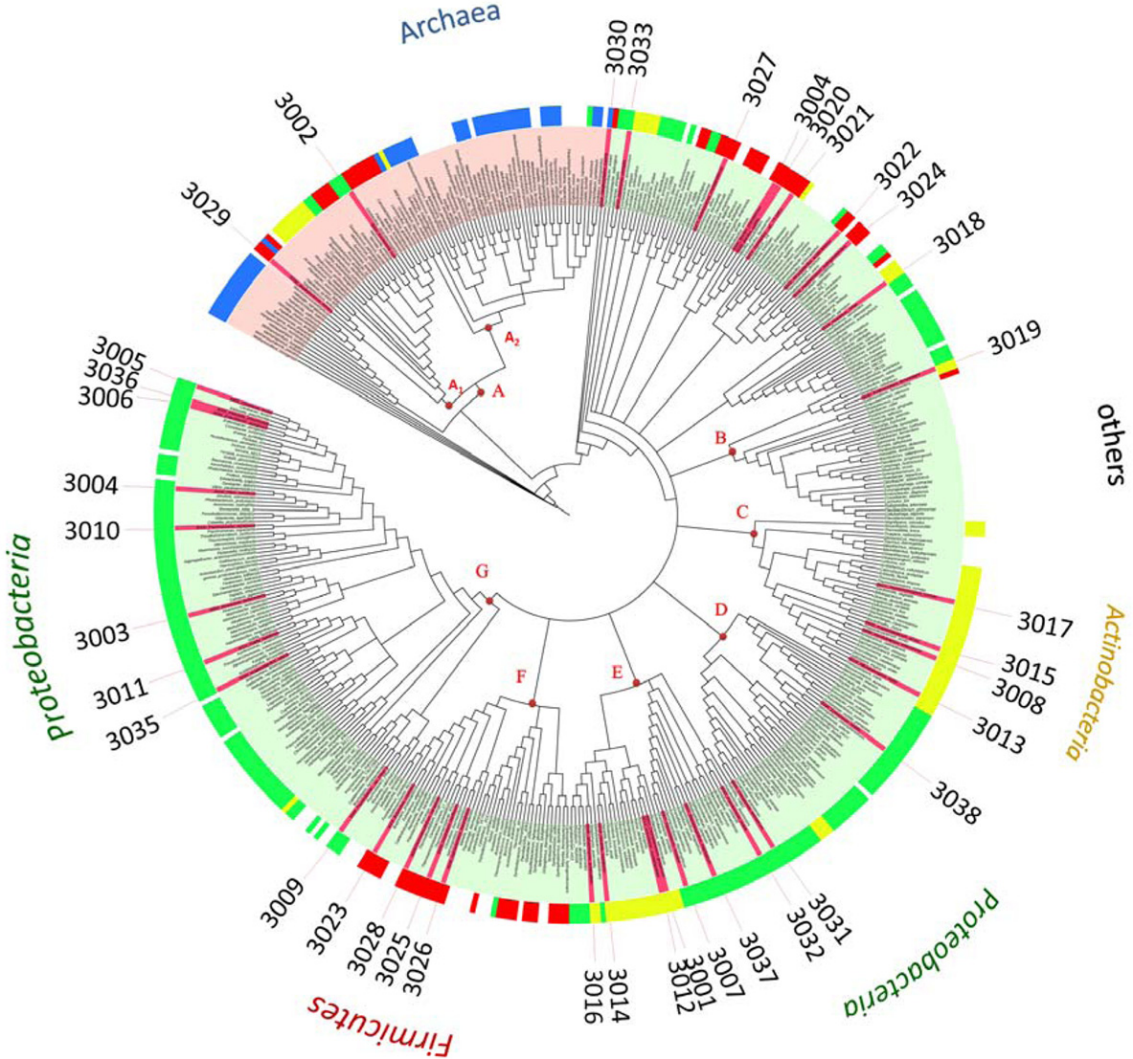
**Circle phylogenetic tree of 459 DHQases, constructed with Maximum Likelihood method with 1000 bootstrap replications.** The salmon color range covers all species with type I DHQases, the light green range covers all species with type II DHQases. The codes for the 38 selected DHQases were assigned outside circles. For the outmost color ring, colored strips indicate the origins (at phylum levels) of the DHQases: *Firmicutes* is marked with red strips, *Proteobacteria* is green, *Actinobacteria* is yellow. All archaea including *Thaumarchaeota*,*Crenarchaeota*, and *Euryarchaeota* are showed in dark blue. Species from other phylum is left with no color strips. Capital letters A-G indicated7 big clades with bootstrap support values >70%.

To display the phylogenetic relationship of all 459 DHQase sequences and taxonomic distribution, their amino acid sequences were used to construct a phylogenetic tree (Figure 1). As seen from Figure 1, seven clades (from cluster A to G) of DHQases were recognized. The majority of type I DHQases fell into the clade A, which contained two sub-clades A_1_ and A_2_. Sub-clade A_1_ covered bacterial type I DHQases from *Proteobacteria, Actinobacteria,* and *Firmicutes*. Sub-clade A_2_ was mainly archaeal DHQases from *Thaumarchaeota*, *Crenarchaeota*, and *Euryarchaeota*. Clades B through G was type II DHQases of different bacterial phyla. DHQases of the clades C and F were mainly from *Actinobacteria* and *Firmicutes*, respectively. DHQases of clades D and G were mainly from *Proteobacteria*. DHQases of clades B and E were from *Proteobacteria*, *Actinobacteria* and other bacterial phyla.

### Cloning and expression of customized DHQase genes in *E. coli* cells

From all retrieved 459 DHQases, 38 were further selected (Table 2).The selection was carried out according to the following criterions: 1) Both type I and type II DHQases were included, and the majority was bacterial type II DHQases; 2) Host genomes harbored a complete shikimate pathway, or taxonomically representative (*Archaea*, *Actinobacteria*, *Firmicutes*, and *Proteobacteria*), or from unique habitats. From the 38 DHQases, 35 were of type II, and 3 were of type I. The theoretical molecular masses of type II DHQases were ranged from 15 to 18 kDa, and their amino acid sequence identities to DHQase from *C. glutamicum* were 41.7-71.9%. The selected DHQases were recorded by referring the codon usages of *E. coli* and of *C. glutamicum* (Nakamura 2007). The new DNA sequences were edited by curing restriction endonucleases (*Nde*I, *Xho*I, *Eco*RI, *Not*I, *Xba*I, *Spe*I and *Pst*I) sites and any predicted secondary mRNA structures. Further, the sequences were customized for “Biobricks” (Knight 2003) by attaching the prefix5′-GCAGAATTCGCGGCCGCTTCTAGA-3′ and the suffix 5′-ACTAGTAGCGGCCGCCTGCAG-3′.

**Table 2:** **Selected DHQase, their origins and theoretical molecular masses of translational products**

**Codes for DHQases** ^**a**^	**Origins**	**NCBI accession numbers of**	**Accession IDs at ** www.genoportal.org	**Theoretical molecular mass (kDa)**	**Sequence identity**
**Type I**					
3002	*Escherichia coli*	YP_489955.1	SBB_00449	27.5	100%
3029	*Staphylococcus aureus*	YP_005744190.1	SBB_00476	26.9	30.0%
3030	*Metallosphaera cuprina*	YP_004409005.1	SBB_00477	23.7	23.5%
**Type II**					
3001	*Corynebacteriumglutamicum*	YP_224725.1	SBB_00448	15.4	100.0%
3003	*Alcanivorax borkumensis*	YP_693728.1	SBB_00450	17.5	50.7%
3004	*Bacillus pseudofirmus*	YP_003425282.1	SBB_00451	16.5	50.0%
3005	*Citrobacter koseri*	YP_001456151.1	SBB_00452	16.5	53.4%
3006	*Enterobacter aerogenes*	YP_004591144.1	SBB_00453	16.5	53.0%
3007	*Gluconobacter oxydans*	YP_190876.1	SBB_00454	17.4	51.1%
3008	*Mycobacterium tuberculosis*	YP_003031384.1	SBB_00455	15.8	52.6%
3009	*Psychrobacter*sp. PRwf-1	YP_001279670.1	SBB_00456	18.4	52.8%
3010	*Psychromonas ingrahamii*	YP_944417.1	SBB_00457	16.5	53.0%
3011	*Xylella fastidiosa*	NP_297340.2	SBB_00458	16.7	52.2%
3012	*Arthrobacter crystallopoietes*	EMY34512.1	SBB_00459	16.3	71.9%
3013	*Micromonas poralupini*	CCH17589.1	SBB_00460	15.4	47.8%
3014	*Nocardia brasiliensis*	YP_006813099.1	SBB_00461	15.6	64.0%
3015	*Propionibacterium acnes*	YP_056375.1	SBB_00462	15.8	43.7%
3016	*Streptomyces acidiscabies*	WP_010352491.1	SBB_00463	16.8	61.4%
3017	*Thermomonas poracurvata*	YP_003300530.1	SBB_00464	16.6	45.3%
3018	*Atopobium rimae*	EEE18006.1	SBB_00465	15.5	41.7%
3019	*Acidimicrobium ferrooxidans*	YP_003110305.1	SBB_00466	16.4	45.8%
3020	*Bacillus subtilis*	NP_390327.1	SBB_00467	16.4	49.3%
3021	*Geobacillus* sp. Y4.1MC1	YP_003988579.1	SBB_00468	16.0	48.6%
3022	*Alicyclobacillus acidocaldarius*	EED07485.1	SBB_00469	16.3	49.6%
3023	*Butyrivibrio crossotus*	EFF68279.1	SBB_00470	22.8	47.1%
3024	*Halanaerobium hydrogeniformans*	YP_003995185.1	SBB_00471	16.6	51.4%
3025	*Clostridium clariflavum*	YP_005045936.1	SBB_00472	15.5	53.1%
3026	*Natranaerobius thermophilus*	YP_001917881.1	SBB_00473	15.8	43.7%
3027	*Ruminococcus champanellensis*	YP_007829441.1	SBB_00474	16.1	49.6%
3028	*Thermodesulfobium narugense*	YP_004438087.1	SBB_00475	16.8	46.3%
3031	*Comamonas testosteroni*	YP_003279351.1	SBB_00478	15.9	52.1%
3032	*Pseudomonas putida*	NP_745147.1	SBB_00479	16.2	59.4%
3033	*Halomonas elongata*	YP_003899044.1	SBB_00480	15.5	55.8%
3034	*Vibrio vulnificus*	NP_935927.1	SBB_00481	18.7	55.6%
3035	*Acidithiobacillus caldus*	YP_004750222.1	SBB_00482	16.9	50.7%
3036	*Klebsiella pneumoniae*	EMH96211.1	SBB_00483	16.5	51.5%
3037	*Magnetospirillum magneticum*	YP_422069.1	SBB_00484	14.5	50.4%
3038	*Zymomonas mobilis*	YP_162472.1	SBB_00485	15.8	52.9%

The amino acid sequence identities of DHQases to the one from *C. glutamicum* (type II) or from *E. coli* (type I) were calculated based on alignment with BLAST searches. The NCBI numbers refer to the wildtype DHQases, and the customized DNA sequences of these DHQases are accessible at http://www.genoportal.org/bbdb.

^a^The first digit of the code represents DHQase catalyzes the 3rd step of shikimate pathway, and the following digits are the order when that DHQases were selected.

All 38 DHQase genes were cloned with *E. coli*. Except those DHQases of *A. ferrooxidans*, *B. pseudofirmus*, *S. aureus* and *Z. mobilis* that occurred as either inclusion bodies or no synthesis in *E. coli* cells, all other DHQase were synthesized and purified. The purified DHQases actively catalyzed the conversion of DHQ into 3-dehydroshikimic acid.

### DHQases have broad ranges of kinetic parameters

**Figure 2:**
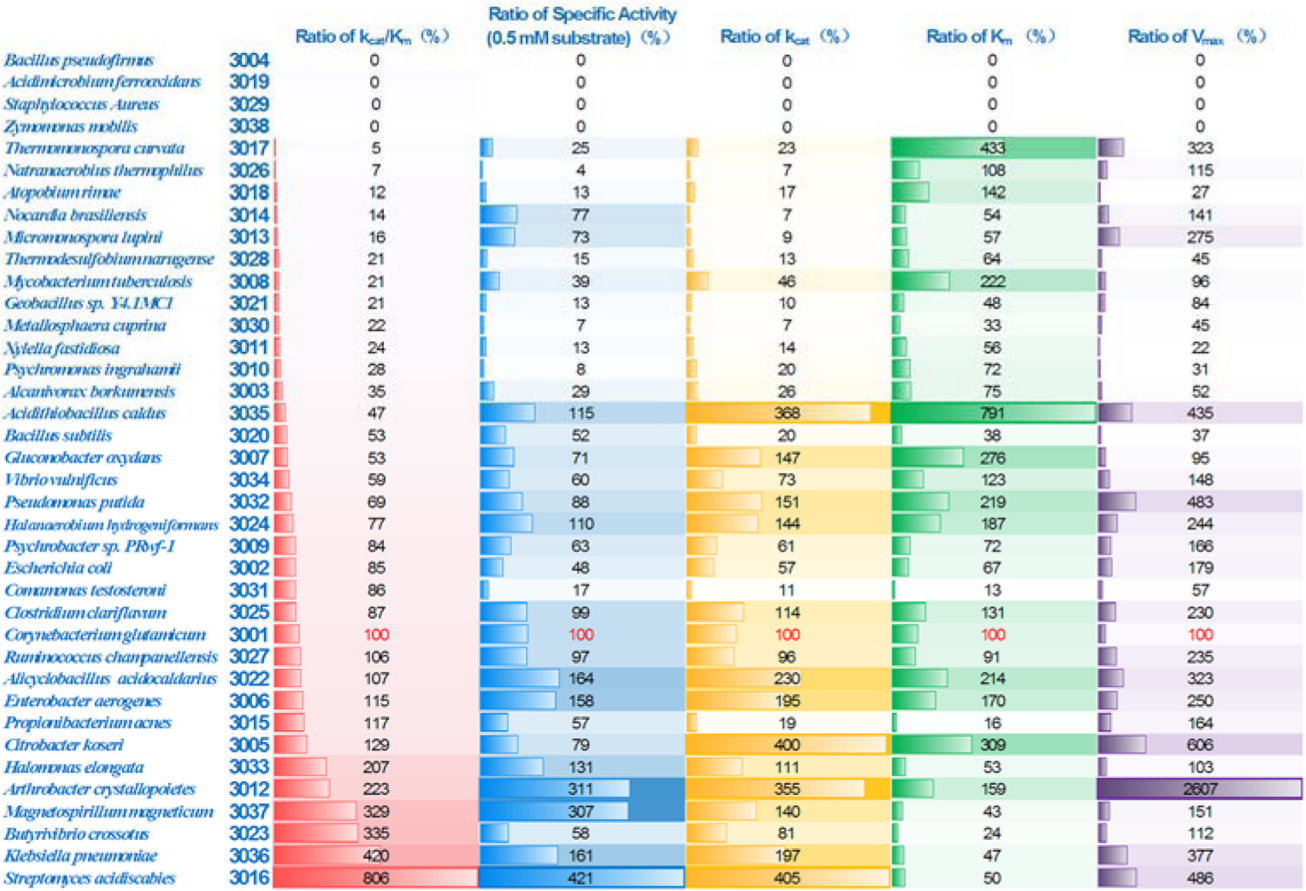
**Demonstration of kinetic diversity of DHQases.** Kinetic parameters were shown as ratio (in %) of value of any DHQase to that of code 3001. Four sets of kinetic parameters were displayed with color gradients.

Catalytic properties such as V_max_, K_m_, k_cat_, and k_cat_/K_m_ of the selected DHQases were determined and are listed in Table 3. Results showed that their kinetic parameters are distributed in broad ranges, which suggested a kinetic diversity of DHQases regarding catalytic capacity and efficiency, and substrate affinity. The V_max_ values of DHQases at the given enzyme concentrations in this study were determined to be 0.57-66.7 μmol/L/s. The K_m_ values were determined to be 37.2-2226.5 μmol/L. These results indicated that natural DHQases had evolved broad catalytic properties, probably for reasons to serve their hosts at different growing conditions. The DHQase (Code 3012) from *A. crystallopoietes* showed the highest catalytic capacity (V_max_ = 66.7 μmol/L/s) and moderate affinity to 3-dehydroquinate (K_m_ = 448.5 μmol/L). The DHQase (Code 3031) from *C. testosteroni* showed highest affinity to 3-dehydroquinate (K_m_ = 37.2 μmol/L) among the tested DHQases. The kinetic parameter k_cat_ signifying the catalytic efficiency of an enzyme, and it was found that the DHQase (Code 3016) from *S. acidiscabies* was the most efficient one among the tested DHQases and its k_cat_ value was determined to be 211.83 s^−1^. When all the tested DHQases were evaluated by k_cat_/K_m_ values, the DHQase (Code 3016) had the highest k_cat_/K_m_value (1.50 L/μmol/s) that represented the best catalytic specificity and efficiency among all the tested DHQases. In addition, the specific activities of DHQases at 3-dehydroquniate concentration of 0.5 mM were determined and DHQase (Code 3016) from *S. acdiscabies* showed the highest activity (501.87 units/mg). Figure 2 depicts a whole image of kinetic parameter distribution of DHQases. Apparently, the kinetic parameters of all tested DHQases varied significantly, reflecting a long-span distribution of their catalytic properties and kinetic diversity.

**Table 3:** **The kinetic parameters of 3-dehydroquinate dehydratases**

Codes for DHQases*	**V**_**max**_ **(μmol/L/s)**	**K**_**m**_ **(μmol/L)**	**k**_**cat**_ **( s** ^**−1**^ **)**	**k**_**cat**_ **/K**_**m**_ **(L/μmol/s)**	Specific Activity** (U/mg)
3001	2.56	281.43	52.26	0.19	119.14
3002	4.57	187.68	29.53	0.16	57.34
3003	1.34	210.71	13.53	0.06	34.72
3004	——	——	——	——	——
3005	15.50	870.37	208.96	0.24	93.91
3006	6.39	478.67	102.15	0.21	187.69
3007	2.43	777.75	77.06	0.10	84.95
3008	2.46	624.52	23.96	0.04	46.37
3009	4.24	203.24	31.67	0.16	75.06
3010	0.80	202.55	10.59	0.05	9.39
3011	0.57	158.79	7.15	0.05	15.06
3012	66.67	448.50	185.43	0.41	369.99
3013	7.04	159.89	4.63	0.03	86.50
3014	3.60	151.51	3.83	0.03	91.93
3015	4.18	46.14	10.00	0.22	67.71
3016	12.42	141.53	211.83	1.50	501.87
3017	8.25	1218.56	11.92	0.01	30.19
3018	0.68	400.82	9.06	0.02	15.64
3019	——	——	——	——	——
3020	0.96	107.07	10.46	0.10	62.27
3021	2.16	133.97	5.33	0.04	15.90
3022	8.26	601.64	119.98	0.20	195.81
3023	2.86	67.88	42.20	0.62	68.99
3024	6.23	526.07	75.11	0.14	131.08
3025	5.89	368.46	59.61	0.16	117.89
3026	2.95	304.54	3.80	0.01	5.16
3027	6.00	255.54	50.20	0.20	116.07
3028	1.16	180.72	6.88	0.04	17.66
3029	——	——	——	——	——
3030	1.14	92.76	3.86	0.04	8.58
3031	1.45	37.16	5.95	0.16	19.82
3032	12.35	617.28	78.87	0.13	105.18
3033	2.64	150.25	57.84	0.38	155.93
3034	3.78	345.90	37.97	0.11	71.87
3035	11.14	2226.50	192.27	0.09	136.57
3036	9.64	131.63	102.70	0.78	192.10
3037	3.87	119.97	73.18	0.61	365.73
3038	——	——	——	——	——

*The first digit of the code represents DHQase catalyzes the 3rd step of shikimate pathway, and the following digits are the order when that DHQase was selected. **Specific activities were determined at substrate concentration of 0.5 mM. “-” indicated no activity was detected.

## Discussion

Kinetic diversity of enzymes is the results of natural revolution in life, similar to their phylogenetic diversity (Zhi et al. 2014). But the kinetic diversity of enzymes had not yet been explored before. This study took DHQase as an example to explore the kinetic diversity of enzymes involved in shikimate pathway. The results from this study demonstrated that DHQases from various hosts had very broad ranges of affinity (K_m_) to substrate, catalytic efficiency and catalytic capacity (k_cat_ and V_max_). It was considered that other enzymes of the shikimate pathway would also have broad ranges of kinetic constants. Such broad ranges of kinetic constants are reflections of natural evolution and adaptation of enzymes, and founded the cornerstones of diverse metabolic fluxes in different lives. Therefore, it would be theoretically feasible to engineer metabolic processes quantitatively by mining enzymatic kinetic database. Such strategy is basically different from the current used strategy, ie., manipulation at levels of genetic transcription and translation (Rytter et al. 2014; Salis et al. 2009; Sohoni et al. 2014).

Recently, synthetic biology has emerged as a new discipline to manipulate biological systems for application and to understand the law of life (Andrianantoandro et al. 2006; Cheng and Lu 2012). The basic units for synthetic biology are standardized biological modules and parts, and standardized enzymes serve as catalytic parts for assembling novel and artificially designed biological systems (Cooling et al. 2010). Such catalytic part library is represented by the chemically synthesized genes of methyl halide transferases (Bayer et al. 2009). In this study, 38 selected DHQase genes were chemically synthesized and customized as Biobricks for future applications. All DHQases have Biobrick adapters, so they are compatible to other Biobrick parts/modules. Our study is part of an on-going project of design and construction of artificial modules for shikimate pathway using synthetic biological tools, aiming to create modules for shikimate pathway that would stimulate industrial applications for bioproduction of aromatic-related primary and secondary compounds. The pipeline for DHQase sequence design, chemical synthesis and purification established from this study has been generalized for other enzymes of the shikimate pathway, and construction of a catalytic part library of shikimate pathway is in progress.

Previous reports described that type I DHQases are represented by DHQases from plants and fungi (Hawkins et al. 1993; Weaver and Herrmann 1997), we have found that all 43 archaeal DHQases in this study were belonging to type I, although this study was focused on type II DHQases. Type II DHQases had been classified into two groups in terms of their kinetic constants k_cat_ (Pan et al. 2012). The first group included DHQase from *S. coelicolor* and had relative high k_cat_ values larger than 100 s^−1^, whereas the sencond group included DHQase from *M. tuberculosis* with a k_cat_ lower than 10 s^−1^. As demonstrated in this study, the k_cat_ values of DHQases distributed contineouslyfrom 4 to over 200 s^−1^, which rendered it unrealistic to devide them into two groups based on k_cat_ values.

Alignments of type II DHQases revealed a number of conserved amino acid residues that are potentially important for catalytic efficiency and capacity. Specifically, the Tyr24 residue facilitated the proton abstraction from substrate, and then the His101 residues promoted the dehydrogenation by donating proton to 1-hydroxyl on C1 of substrate as a general acid (Blomberg et al. 2009; Pan et al. 2012). The residue His101was found in all the type II DHQases. But Tyr24 was replaced by Phe residue in DHQase from *B. subtilis* (Code 3020) (Data not shown). It was found that this DHQase (Code 3020) were functional and catalyzed the conversion of DHQ to 2-dehydroshikimate. The Phe residue was found in eighteen *B. subtilis* genomes as well as other bacilli genomes such as *B. mojavensis*, *B. tequilensis*, *B. vallismortis*, *B. atrophaeus*, demonstrating a natural evolution of Tyr24 into a Phe24 residue in members of the genus *Bacillus*. This natural evolution resulted in a low efficient but still active DHQase, which might be a result of adaption to the *in vivo* metabolic fluxes of bacilli cells. The catalytic mechanism of the Phe-DHQase has not been explored. Since Phe residue is rather stable and hard to be deprotonated, the catalytic mechanism of the Phe-DHQases might be different from that of the previous characterized DHQases.
